# Comparative chromosomal mapping of microsatellite repeats reveals divergent patterns of accumulation in 12 Siluridae (Teleostei: Siluriformes) species

**DOI:** 10.1590/1678-4685-GMB-2020-0091

**Published:** 2020-11-06

**Authors:** Sukhonthip Ditcharoen, Francisco de Menezes Cavalcante Sassi, Luiz Antonio Carlos Bertollo, Wagner Franco Molina, Thomas Liehr, Pasakorn Saenjundaeng, Alongklod Tanomtong, Weerayuth Supiwong, Chatmongkon Suwannapoom, Marcelo de Bello Cioffi

**Affiliations:** 1 Khon Kaen University Khon Kaen University Department of Biology MuangKhon Kaen Thailand Khon Kaen University, Faculty of Science, Department of Biology, Toxic Substances in Livestock and Aquatic Animals Research Group, Muang, Khon Kaen, Thailand.; 2 Universidade Federal de São Carlos (UFSCar) Universidade Federal de São Carlos (UFSCar) Departamento de Genética e Evolução São CarlosSP Brazil Universidade Federal de São Carlos (UFSCar), Departamento de Genética e Evolução, São Carlos, SP, Brazil.; 3 Universidade Federal do Rio Grande do Norte Universidade Federal do Rio Grande do Norte Departamento de Biologia Celular e Genética NatalRN Brazil Universidade Federal do Rio Grande do Norte (UFRN), Centro de Biociências, Departamento de Biologia Celular e Genética, Natal, RN, Brazil.; 4 University Hospital Jena University Hospital Jena Institute of Human Genetics Jena Germany University Hospital Jena, Institute of Human Genetics, Jena, Germany.; 5 Khon Kaen University Khon Kaen University Faculty of Applied Science and Engineering MuangNong Khai Thailand Khon Kaen University, Faculty of Applied Science and Engineering, Nong Khai Campus, Muang, Nong Khai, Thailand.; 6 University of Phayao University of Phayao Department of Fishery School of Agriculture and Natural Resources Muang Phayao Thailand University of Phayao, School of Agriculture and Natural Resources, Department of Fishery, Muang Phayao, Thailand.

**Keywords:** Repetitive DNAs, fish, chromosomal rearrangements, karyotype evolution

## Abstract

The freshwater family Siluridae occurs in Eurasia and is especially speciose in South and Southeast Asia, representing an important aquaculture and fishery targets. However, despite the restricted cytogenetic data, a high diploid number variation (from 2n=40 to 92) characterizes this fish group. Considering the large genomic divergence among its species, silurid genomes have experienced an enormous diversification throughout their evolutionary history. Here, we aim to investigate the chromosomal distribution of several microsatellite repeats in 12 Siluridae species and infer about their possible roles in the karyotype evolution that occurred in this group. Our results indicate divergent patterns of microsatellite distribution and accumulation among the analyzed species. Indeed, they are especially present in significant chromosome locations, such as the centromeric and telomeric regions, precisely the ones associated with several kinds of chromosomal rearrangements. Our data provide pieces of evidence that repetitive DNAs played a direct role in fostering the chromosomal differentiation and biodiversity in this fish family.

## Introduction

The freshwater family Siluridae ranges in Eurasia, but containing the higher number of species in South and Southeast Asia (Bornbusch, 1995; Kottelat, 2013), with 103 recognized species (Fricke *et al.*, 2019), thus representing important aquaculture and fishery targets. Silurid species show a significant size diversity, such as *Silurus glanis*, reaching over 300 kg in weight and 2m in size (Linhart *et al.*, 2002) and *Silurus soldatovi*, that reaches 400 kg in weight and up to 4m in size (Berg, 1964), while others are much smaller, being used as ornamental fishes (Ng *et al.*, 1994; Chapman *et al.*, 1997; Ng and Ng, 1998;) or biological indicators (Ng and Lim, 1992; Ng and Ng, 1998). The monophyletic status of Siluridae is supported and confirmed by both morphological and molecular data (Bornbusch, 1995; Hardman, 2005), and, altough their phylogenetic position was doubtful for many years (Bornbusch, 1995; Hardman, 2005; Sullivan *et al.*, 2006), a recentl study placed this family in the root of taxon Siluroidea (Kappas *et al.*, 2016).

Cytogenetical studies in Siluridae are still mostly restricted to conventional cytogenetic protocols, with some exceptions where the molecular cytogenetic approach has been used (Verma *et al.*, 2011; Ditcharoen *et al.*, 2019). However, despite the restricted cytogenetic data, a high 2n variation characterizes this fish group, ranging from 40 in *Silurichthys phaiosoma* (Ditcharoen *et al.*, 2019) to 92 in *Kryptopterus cryptopterus* (Donsakul and Magtoon, 1996) and *Kryptopterus geminus* (Ditcharoen *et al.*, 2019). It is also known that *Phalacronotus* is the only genus that maintains the diploid number conservation with 2n = 64 in all analyzed species, while other genera in this family display a substantial variation (Ditcharoen *et al.*, 2019). On the mapping of highly repetitive sequences, the high 2n variation also appears to be followed by a large variation of ribosomal DNAs loci among silurid species (Ditcharoen *et al.*, 2019). Considering the extensive genomic reorganization, as revealed by CGH (comparative genomic hybridization), it is evident that silurid genomes have experienced an enormous diversification throughout their evolutionary history (Ditcharoen *et al.*, 2019).

Microsatellites are repetitive DNA sequences, varying from one to six nucleotides, found in genomes of all eukaryotic organisms (Cioffi and Bertollo, 2012; López-Flores and Garrido Ramos, 2012). These repeats can also be associated with coding regions of structural genes and between other repetitive sequences (Tautz and Renz, 1984), contributing to the functional and structural organization of the genome (Schueler *et al.*, 2001). Fish genomes usually have microsatellites distributed throughout telomeric and centromeric regions of autosomal and sex chromosomes, associated with other repetitive DNA sequences (Cioffi and Bertollo, 2012). Additionally, repetitive DNAs have an important role in speciation, differentiation of sex-specific regions, and promotion of biodiversity (Vicari *et al.*, 2005; Cioffi *et al.*, 2009; Sember *et al.*, 2018). Therefore, here we analyzed the chromosomal location several microsatellites repeats to explore the intergenomic divergence at the chromosomal level in 12 Silurid species; the sampling resembles the one previously analyzed by Ditcharoen *et al.* (2019) with different cytogenetic methods. Indeed, our recent result provided new insights into the karyotype differentiation of this fish group, with a better understanding of the chromosomal organization of repetitive DNAs and uncovering chromosome homologies and differences among the studied species.

## Material and Methods

**Figure 1:**
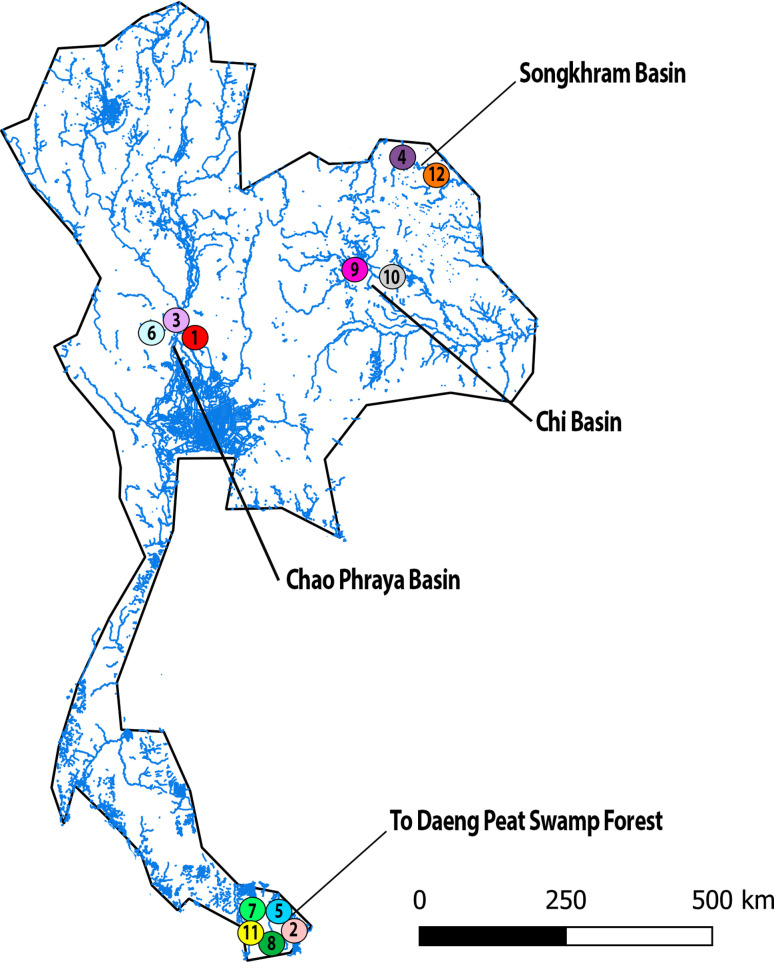
Map of Thailand highlighting the collection sites of Silurid fishes studied herein. The numbers represent the different species sampled as shown in **Table 1.** The map was produced using the software QGis 3.4.4 (https://qgis.org), Inkscape 0.92 (https://inkscape.org), and Adobe Photoshop CC 2015 (San Jose, CA, USA).

Twelve silurid species were collected in the river basins of Thailand **(**Figure 1, Table 1**).** All individuals were deposited in the fish collection of the Cytogenetic Laboratory, Department of Biology, Faculty of Science (Khon Kaen University). The procedures followed ethical protocols and anesthesia was conducted with clove oil before euthanasia, as approved by the Institutional Animal Care and Use Committee of Khon Kaen University, based on the Ethics of Animal Experimentation of the National Research Council of Thailand IACUC-KKU-10/62.

**Table 1:** Species analyzed, collection sites and the number of analyzed individuals (n).

Species	Locality	n
1. *Belodontichthys truncatus*	Chao Phraya Basin	04f; 04m
	14°52’17.3“N 100°24’32.3”E	
	Ton Pho, Mueang Sing Buri District, Sing Buri	
2. *Kryptopterus bicirrhis*	To Daeng peat swamp forest	07f; 08m
	6°04’34.0“N 101°57’46.0”E	
	Puyo, Su-ngai Kolok District, Narathiwat	
3. *Kryptopterus geminus*	Chao Phraya Basin	08f; 11m
	14°52’17.3“N 100°24’32.3”E	
	Ton Pho, Mueang Sing Buri District, Sing Buri	
4. *Kryptopterus limpok*	Songkhram Basin	07f; 10m
	17°59’37.8“N 103°26’54.2”E	
	Dong Mo Thong Tai, Ban Muang District, Sakon Nakhon	
5. *Kryptopterus macrocephalus*	To Daeng peat swamp forest	06f; 06m
	6°04’34.0“N 101°57’46.0”E	
	Puyo, Su-ngai Kolok District, Narathiwat	
6. *Micronema cheveyi*	14°52’17.3“N 100°24’32.3”E	09f; 10m
	Ton Pho, Mueang Sing Buri District, Sing Buri	
7. *Ompok fumidus*	To Daeng peat swamp forest	05f; 07m
	6°04’34.0“N 101°57’46.0”E	
	Puyo, Su-ngai Kolok District, Narathiwat	
8. *Ompok siluroides*	To Daeng peat swamp forest	04f; 05m
	6°04’34.0“N 101°57’46.0”E	
	Puyo, Su-ngai Kolok District, Narathiwat	
9. *Phalacronotus apogon*	Chi Basin	06f; 05m
	16°13’35.5“N 103°19’30.6”E	
	Tha Khon Yang, Kantharawichai District, Maha Sarakham	
10. *Phalacronotus bleekeri*	Chi Basin	07f; 04m
	16°13’35.5“N 103°19’30.6”E	
	Tha Khon Yang, Kantharawichai District, Maha Sarakham	
11. *Silurichthys phaiosoma*	To Daeng peat swamp forest	04f; 06m
	6°04’34.0“N 101°57’46.0”E	
	Puyo, Su-ngai Kolok District, Narathiwat	
12. *Wallago attu*	Songkhram Basin	03f; 04m
	17°59’37.8“N 103°26’54.2”E	
	Dong Mo Thong Tai, Ban Muang District, Sakon	

Chromosomes were obtained by the classical air-drying method from kidney cells (Bertollo *et al.*, 2015). The preparations were then dropped onto clean glass slides at 55 °C and stained with Giemsa 5%. The hybridization procedure was taken under high stringency conditions (Yano *et al.*, 2017), with six microsatellites as probes [(CA)_15_, (CAC)_10,_ (CAT)_10_, (GC)_15_, (CGG)_10_, (A)_30_] directly labeled with Cy-3 during the synthesis (Kubat *et al.*, 2008). These sequences were selected from a pool of microsatellite repeats since they are commonly accumulated in several fish genomes (e.g. Nanda *et al.*, 1990; Vanzela *et al.*, 2002; Martins, 2007; Cioffi *et al.*, 2011; Cioffi and Bertollo, 2012; Poltronieri *et al.*, 2014; Cioffi *et al.*, 2015; Pucci *et al.*, 2016; Ráb *et al.*, 2016; Sassi *et al.*, 2019; Supiwong *et al.*, 2019)

We performed at least three repetitions for each experiment and analyzed at least 30 metaphases per experiment to check the consistency of the results. Images were captured using an Olympus BX50 microscope (Olympus Corporation, Ishikawa, Japan) with CoolSNAP and processed using Image-Pro Plus 4.1 software (Media Cybernetics, Silver Spring, MD, USA).

## Results

**Figure 2:**
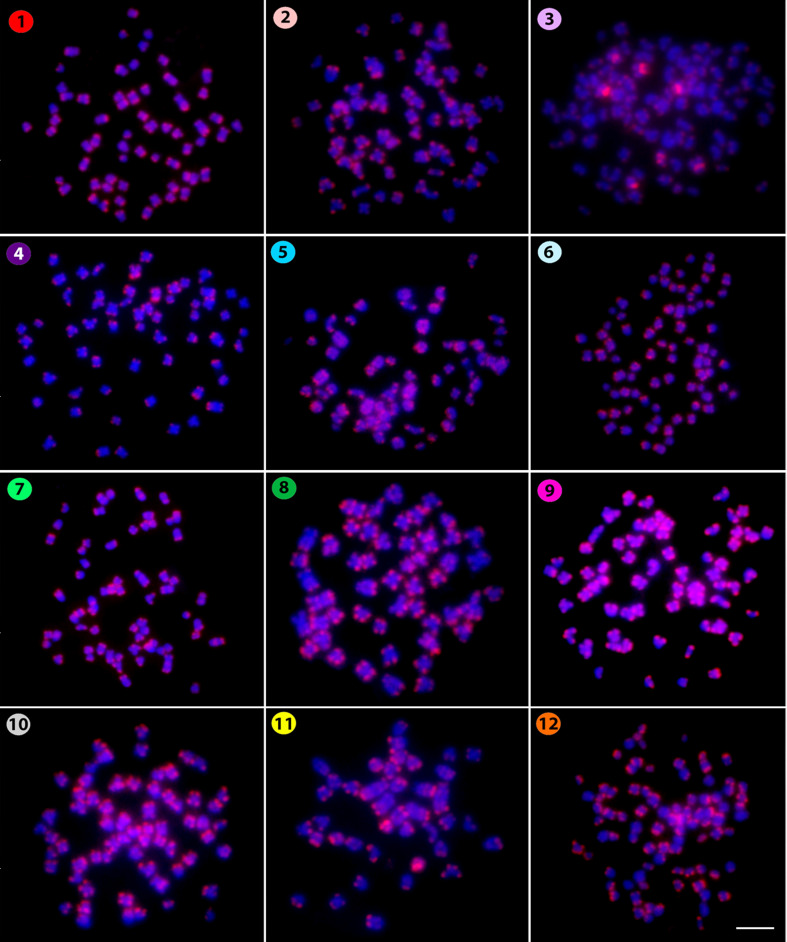
Hybridization pattern of the (CA)_15_ microsatellite probe (in red) on metaphase chromosomes of *Belodontichthys truncates* (**1**); *Kryptopterus bicirrhis* (**2**); *Kryptopterus geminus* (**3**); *Kryptopterus limpok* (**4**); *Kryptopterus macrocephalus* (**5**); *Micronema chevevi* (**6**); *Ompok fumidus* (**7**); *Ompok siluroides* (**8**); *Phalacronotus apogon* (**9**); *Phalacronotus bleekeri* (**10**); *Silurichthys phaiosoma* (**11**) and *Wallago attu* (**12**). Scale bar = 5 μm.

**Figure 3:**
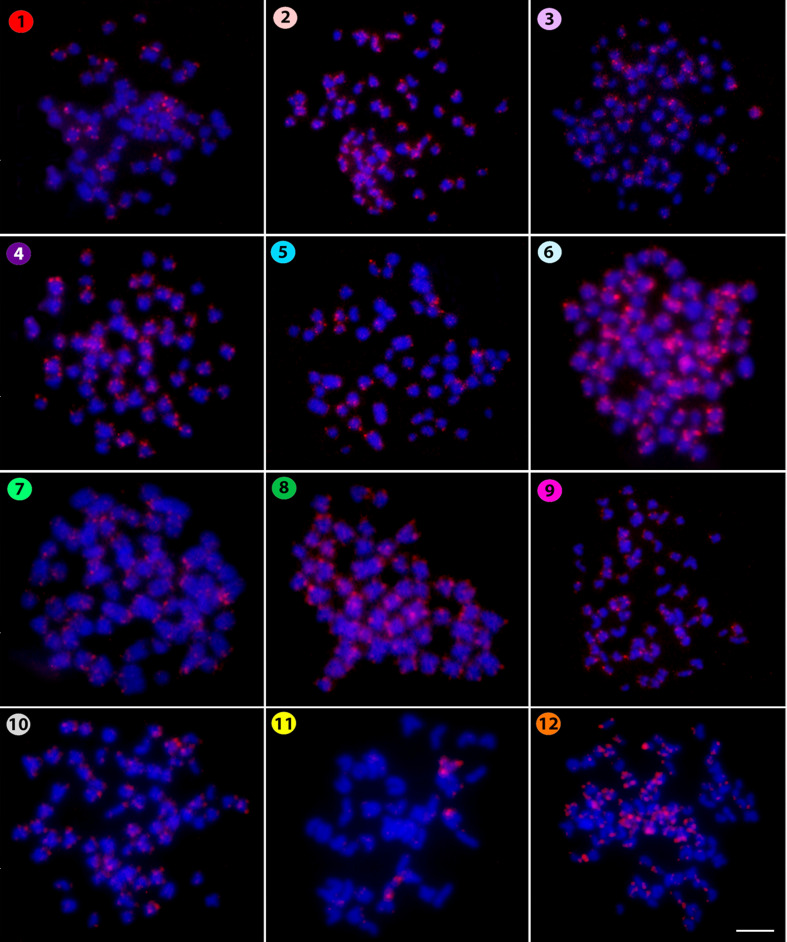
Hybridization pattern of the (CAC)_10_ microsatellite probe (in red) on metaphase plates of *Belodontichthys truncates* (**1**); *Kryptopterus bicirrhis* (**2**); *Kryptopterus geminus* (**3**); *Kryptopterus limpok* (**4**); *Kryptopterus macrocephalus* (**5**); *Micronema chevevi* (**6**); *Ompok fumidus* (**7**); *Ompok siluroides* (**8**); *Phalacronotus apogon* (**9**); *Phalacronotus bleekeri* (**10**); *Silurichthys phaiosoma* (**11**) and *Wallago attu* (**12**). Scale bar = 5 μm.

**Figure 4:**
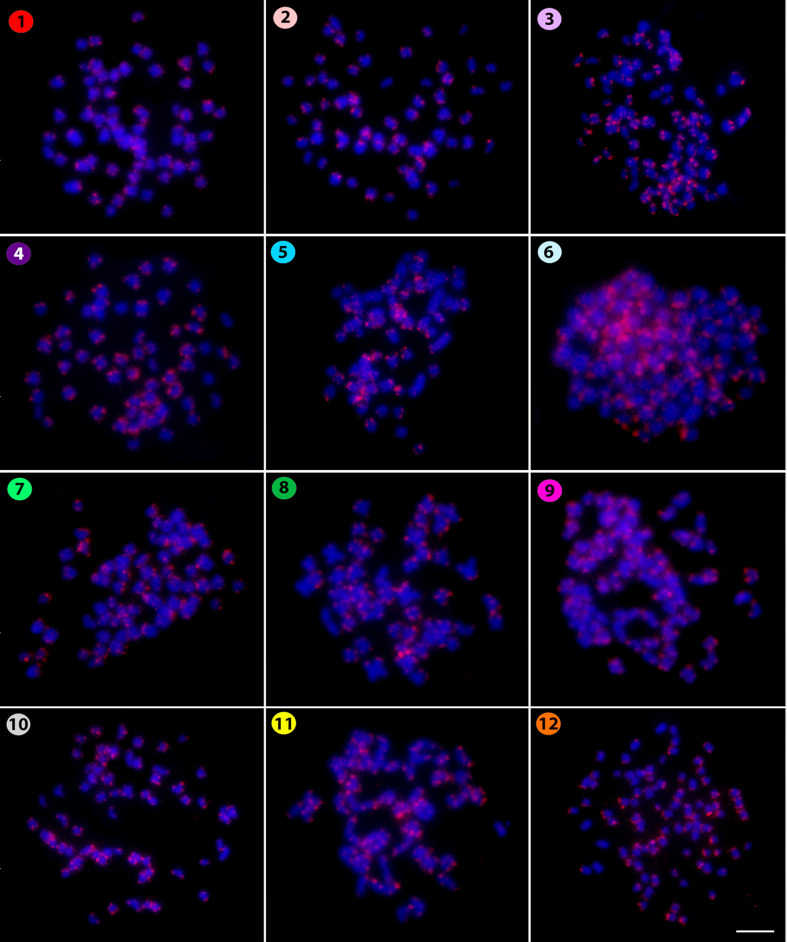
Hybridization pattern of the (CAT)_10_ microsatellite probe (in red) on metaphase plates of *Belodontichthys truncates* (**1**); *Kryptopterus bicirrhis* (**2**); *Kryptopterus geminus* (**3**); *Kryptopterus limpok* (**4**); *Kryptopterus macrocephalus* (**5**); *Micronema chevevi* (**6**); *Ompok fumidus* (**7**); *Ompok siluroides* (**8**); *Phalacronotus apogon* (**9**); *Phalacronotus bleekeri* (**10**); *Silurichthys phaiosoma* (**11**) and *Wallago attu* (**12**). Scale bar = 5 μm.

**Figure 5:**
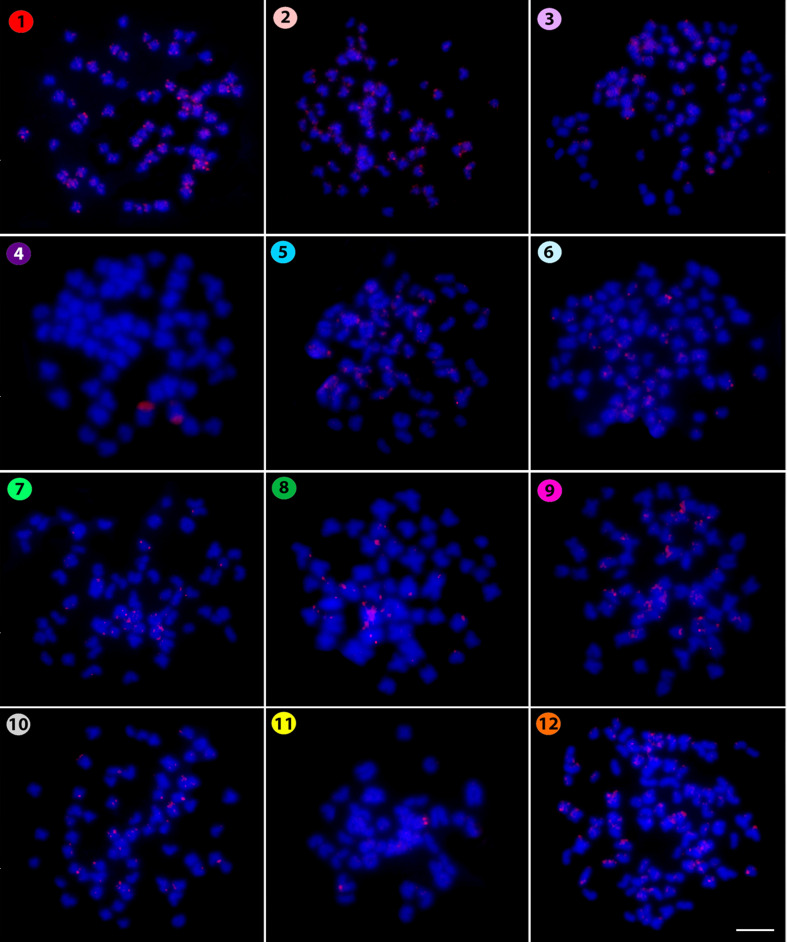
Hybridization pattern of the (GC)_15_ microsatellite probe (in red) on metaphase plates of *Belodontichthys truncates* (**1**); *Kryptopterus bicirrhis* (**2**); *Kryptopterus geminus* (**3**); *Kryptopterus limpok* (**4**); *Kryptopterus macrocephalus* (**5**); *Micronema chevevi* (**6**); *Ompok fumidus* (**7**); *Ompok siluroides* (**8**); *Phalacronotus apogon* (**9**); *Phalacronotus bleekeri* (**10**); *Silurichthys phaiosoma* (**11**) and *Wallago attu* (**12**). Scale bar = 5 μm.

**Figure 6:**
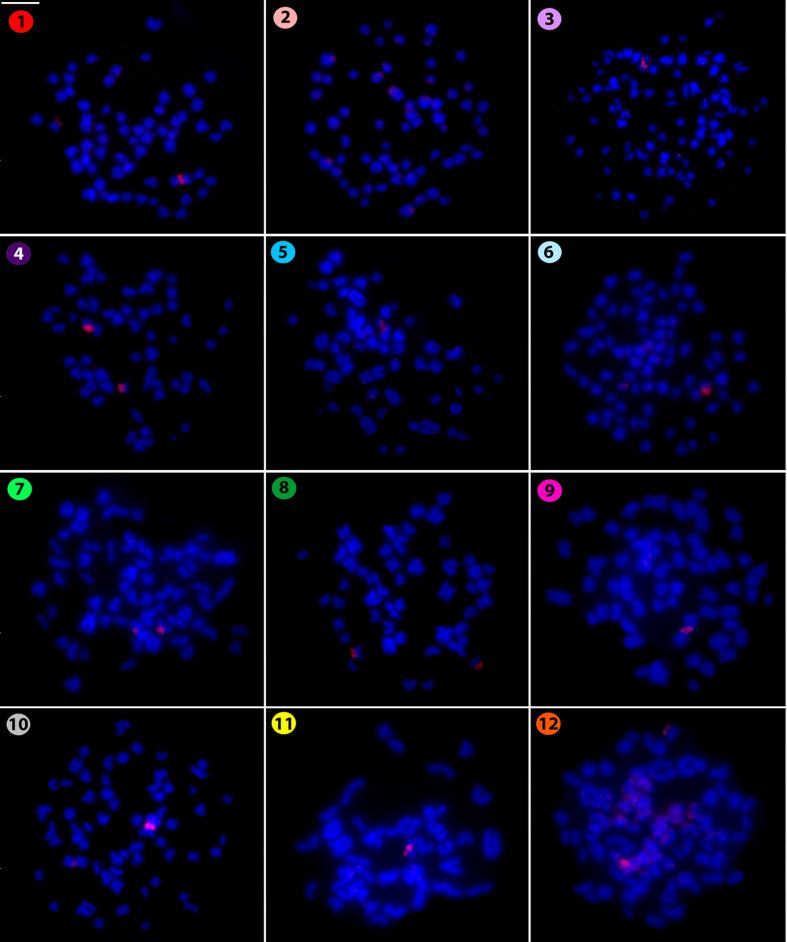
Hybridization pattern of the (CGG)_10_ microsatellite probe (in red) on metaphase plates of *Belodontichthys truncates* (**1**); *Kryptopterus bicirrhis* (**2**); *Kryptopterus geminus* (**3**); *Kryptopterus limpok* (**4**); *Kryptopterus macrocephalus* (**5**); *Micronema chevevi* (**6**); *Ompok fumidus* (**7**); *Ompok siluroides* (**8**); *Phalacronotus apogon* (**9**); *Phalacronotus bleekeri* (**10**); *Silurichthys phaiosoma* (**11**) and *Wallago attu* (**12**). Scale bar = 5 μm.

The microsatellite (CA)_15_ revealed a telomeric pattern of accumulation in all chromosomes of all species (Figure 2), except for *Kryptopterus geminus*, where small telomeric signals occured in addition to strong centromeric ones in some other chromosomes. Similarly, the microsatellite (CAC)_10_ also had a telomeric distribution on chromosomes (Figure 3), but again with an exception, in this case for *Silurichthys phaiosoma* which had only strong centromeric and telomeric signals in two acrocentric pairs, a larger and a smaller one, respectively. However, separate scattered signals were also observed in several other chromosomes. As to the microsatellite (CAT)_10_, all species have scattered telomeric hybridization signals (Figure 4). In turn, a very diverse distribution pattern was observed for the microsatellite (GC)_15_ (Figure 5), where a dispersed distribution of small signals occurred in all chromosomes of *Belodontichthys truncatus*, *Kryptopterus bicirrhis*, *K. geminus* and *K. macrocephalus*. However, in *Micronema cheveyi*, *Ompok fumidus*, *O*. *siluroides*, *Phalacronotus apogon*, *P. bleekeri,* and *Wallago attu*, hybridization signals occurred in the centromeric and telomeric regions of almost half chromosomes of the complement. Yet, in *Kryptopterus limpok* and *Silurichthys phaiosoma* only a single pair of chromosomes were labeled in the centromeric region with such probe. The microsatellite (CGG)_10_ had a very contrasting distribution compared to the other microsatellites. In this case, only one chromosome pair has telomeric signals in the p arms, in all twelve species analyzed (Figure 6). The microsatellite (A)_30_ was the only one not found in any of the examined species (data not shown).

## Discussion

The role of repetitive DNAs in the genome evolution has been documented for different fish groups (Cioffi *et al.*, 2010; Cioffi and Bertollo, 2012; Terencio *et al.*, 2013; Yano *et al.*, 2014; Cioffi *et al.*, 2015; Moraes *et al.*, 2017, 2019; Sassi *et al.*, 2019). Worthy of note is the great evolutionary diversification that Siluriformes fishes have experienced, especially at the chromosomal level. Here, six mono-, bi- and tri-nucleotide microsatellite sequences were mapped on chromosomes of twelve Siluridae species. Except for (A)_30-_, which was not found to occur in any of the analyzed species, all other probes generated ell visible hybridization patterns. However, highly divergent distributions have been found, even among congeneric species, as observed in *Kryptopterus*. Accordingly, this genus displays different 2n, karyotypes and an extensive variation of their repetitive DNA content (Ditcharoen *et al.*, 2019). On the other hand, the *Phalacronotus* species had a similar distribution pattern among chromosomes, probably linked to their chromosomal-conserved characteristics, since they share similarities in both karyotype and genome features (Ditcharoen *et al.*, 2019). It is also remarkable that the same kind of microsatellite did not present the same pattern among silurids. Indeed, very different hybridization patterns for the same microsatellite occur among distinct species, as for the (CA)_15_, (GC)_15_ and (CAC)_10_ probes, for example, although mostly restricted to centromeric and telomeric regions, where a significant fraction of repetitive DNA is localized (Cioffi and Bertollo, 2012).

Additionally, the preferred telomeric and centromeric locations of microsatellites among silurids are, of course, of significance. For instance, this distribution pattern is found in some Siluriformes such as in the Neotropical catfishes *Imparfinis schubarti* (Heptapteridae), *Steindachneridion scriptum* (Pimelodidae), and *Rineloricaria latirostris* (Loricariidae) in which a remarkable accumulation of both (GA)_15_ and (A)_30_ microsatellites in telomeric regions occurs (Vanzela *et al.*, 2002; Supiwong *et al.*, 2014). A similar distribution is also present in the chromosomes of the zebrafish, *Danio rerio*, showing (CA)_n_ and (GT)_n_ repeats clustered in the centromeric and telomeric regions (Shimoda *et al.*, 1999; Supiwong *et al.*, 2014) and the wolffish, *Hoplias malabaricus*, where 12 different microsatellite repeats, including (CA)_15_ and (GA)_15_, showed strong hybridization signals at subtelomeric and heterochromatic regions of several autosomes, in addition to a strong accumulation on the sex chromosomes (Cioffi *et al.*, 2011, Supiwong *et al.*, 2014). In fact, for most of these species, the 18S rDNA repeats are found in the short arms of a single chromosome pair (Ditcharoen *et al.*, 2019), and this region matches the position of the (CGG)n marks found in our experiments. Similarities of both microsatellite and ribosomal DNA location do not seem to be a rare event among fishes, as they are also found in other species, such as *Lebiasina bimaculata* (Sassi *et al.*, 2019) and *Hepsetus odoe* (Carvalho *et al.*, 2017), for example. Indeed, G+C rich motifs are common in exons of all vertebrates (Chistiakov *et al.*, 2006). Since higher recombination rates can be found near the telomeric region (Jensen-Seaman *et al.*, 2004), the physical proximity of microsatellite and rDNA repeats could favor the evolutionary spreading of both sequences together, as triplet sequences are particularly able to stabilize, by hairpin, some alternative structures generated from DNA polymerase slippage (Sinden, 1999). Reinforcing the above considerations, *Silurichthys phaiosoma* has a very particular distribution of the (CAC)n repeats, accumulated in the centromeric and telomeric regions of two acrocentric pairs, respectively. Accordingly, this species also presents a unique pattern of 5S rDNA distribution concerning the other silurids, with the spreading of multiple loci in the karyotype. Besides, a 5S rDNA site is found in the telomeric region of the long arms of the 18^th^ chromosome pair (Ditcharoen *et al.*, 2019), the same one that harbors a conspicuous (CAC)n site.

It is known that eukaryotic centromeres are usually composed of AT-rich DNA (Blackburn and Szostak, 1984) and is commonly rich in heterochromatin, with a complex composition of several repetitive in tandem DNAs (López-Florez and Garrido-Ramos, 2012). Although (AC)n represents the most common microsatellites (Chistiakov *et al.*, 2006), it is noteworthy the predominance of (GC) rich microsatellites in the heterochromatic regions of fishes (Artoni and Bertollo, 1999; Kavalco *et al.*, 2005; Oliveira *et al.*, 2015; Sassi *et al.*, 2019). Accordingly, at least six Siluridae species now investigated (*Micronema cheveyi*, *Ompok fumidus*, *O. siluroides*, *Phalacronotus apogon*, *P. bleekeri,* and *Wallago attu*) have (GC)n pericentromeric signals for almost half chromosomes, in addition to other species, like *Kryptopterus limpok* and *Silurichthys phaiosoma*, that have a single labeled chromosome pair but also in this same region. These findings suggest an association and accumulation of such sequences in this relevant chromosome region, as observed in several other fish species (reviewed in Cioffi and Bertollo, 2012).

Repetitive DNA sequences could act as primary driving forces in speciation (reviewed in Biémont and Vieira, 2006). These sequences are highly associated with heterochromatic regions, thus contributing to gene activation and structural maintenance of chromosomes (Dernburg *et al.*, 1996). Therefore, great variations in the amount and position of these sequences could create fertility barriers by fostering the occurrence of chromosomal rearrangements (Cioffi and Bertollo, 2012). Indeed, the distribution of microsatellite motifs in fish genomes could be biased to some specific noncoding regions, as found in the Asian swamp eel *Monopterus albus* (Li *et al.*, 2017). Additionally, closely related fish species involved in recent speciation events could present a differential pattern in the distribution and quantity of microsatellite sequences on chromosomes, as demonstrated for naked catfishes (Supiwong *et al.*, 2014), channid fishes (Cioffi *et al.*, 2015) and Siluridae species in this paper.

Our results indicate that microsatellite sequences have divergent patterns of distribution and accumulation among Siluridae fishes, probably fostering the chromosomal differentiation and biodiversity in this fish family. Indeed, they are especially present in especific chromosome locations, such as the centromeric and telomeric regions, precisely the ones that are associated with several kinds of chromosomal rearrangements. In addition to their probable roles during chromosomal diversification, it is also highlighted that microsatellites can have a close association with other important classes of repetitive sequences, like ribosomal DNAs. This association can represent a good strategy for increasing biodiversity, facilitating a combined distribution of distinct DNA sequences along with the evolutionary divergence.
